# Tumor suppressor in lung cancer identifies latency infected cells in non malignant HTLV-1 infection

**DOI:** 10.1186/1742-4690-12-S1-O26

**Published:** 2015-08-28

**Authors:** Kiruthika Manivannan, Aileen G Rowan, Graham P Taylor, Charles RM Bangham

**Affiliations:** 1 Section of Virology, Department of Medicine, Imperial College London, London, UK

## 

HTLV-1 Tax protein is usually undetectable in freshly isolated peripheral blood mononuclear cells (PBMCs). After in vitro culture, the provirus is reactivated and Tax protein is detectable only in a proportion of infected CD4+ T cells: the percentage of Tax+ cells is always lower than the proviral load (PVL). To identify and further analyse the latently infected cells we have measured the expression of tumour suppressor of lung cancer 1 (TSLC1). TSLC1 is a member of the immunoglobulin super family that is expressed on all cells with the exception of PBMCs. It mediates cell-to-cell adhesion by either homophilic or heterophilic interactions with other members of the immunoglobulin family and also signals to the actin cytoskeleton. TSLC1 has been shown to be expressed on primary adult T cell leukaemia (ATL) cells and ATL cell lines. We assayed the expression of TSLC1 in the PBMCs of 13 asymptomatic carriers (AC) and 13 HAM/TSP patients by flow cytometry. We found that the percentage of TSLC1+ CD4+ T cells was positively correlated with the PVL (P < 0.0001). To test whether TSLC1+ cells themselves were infected, we flow sorted TSLC1+ CD4+ T cells of 3 ACs and 3 HAM/TSP patients. A median of 95% of TSLC1+ CD4+ T cells carried the provirus. Whilst only 20% of infected CD4+ T cells expressed Tax, a further 47% were identified on the basis of TSLC1 expression. As the host cytotoxic T cell (CTL) response is an important protective factor, we are now testing the hypothesis that the presence of TSLC1 on the surface of infected cells affects its recognition and subsequent killing by CTLs.

